# Environmental and technical impacts of floating photovoltaic plants as an emerging clean energy technology

**DOI:** 10.1016/j.isci.2022.105253

**Published:** 2022-10-04

**Authors:** Hamid M. Pouran, Mariana Padilha Campos Lopes, Tainan Nogueira, David Alves Castelo Branco, Yong Sheng

**Affiliations:** 1 The University of Wolverhampton, Wulfruna St, Wolverhampton, UK; 2 Federal University of Rio de Janeiro, Rio de Janeiro, Brazil

**Keywords:** Applied sciences, Energy sustainability, Green engineering

## Abstract

Floating photovoltaic (FPV) plants present several benefits in comparison with ground-mounted photovoltaics (PVs) and could have major positive environmental and technical impacts globally. FPVs do not occupy habitable and productive areas and can be deployed in degraded environments and reduce land-use conflicts. Saving water through mitigating evaporation and improving water security in arid regions combined with the flexibility for deployment on different water bodies including drinking water reservoirs are other advantages of FPVs. They also have higher efficiency than ground-mounted PV solar and are compatible with the existing hydropower infrastructures, which supports diversifying the energy supply and its resilience. Despite the notable growth of FPVs on an international scale, lack of supporting policies and development roadmaps by the governments could hinder FPVs’ sustainable growth. Long-term reliability of the floating structures is also one of the existing concerns that if not answered could limit the expansion of this emerging technology.

## Graphical abstract

**Figure:**
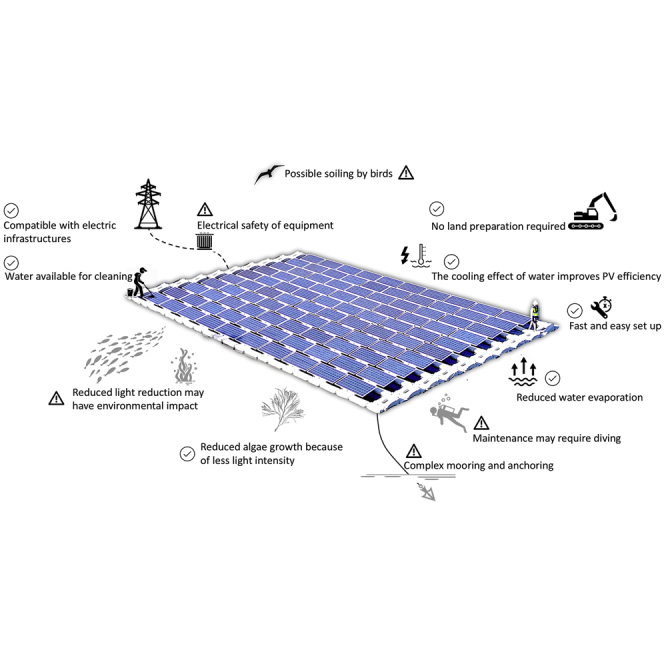

## Introduction

The concept of innovation in renewable energies is often associated with developing cutting-edge technologies. However, innovative solutions for sustainable energy demand are not always complicated. Floating photovoltaics (FPV) farms belong to this group. This perspective article aims to highlight some of the main opportunities and challenges/knowledge gaps of FPV systems as an emerging concept among renewables. In this forward-looking piece, after introducing the concept of FPV solar, some of the socio-environmental impacts of FPVs including job creation, non-occupation of habitable areas, and improving water security are discussed. This is followed by evaluating the technical benefits that emphasizes on the use of degraded areas, reducing algal bloom, improving photovoltaic (PV) panels efficiency, compatibility with hydropower dams, and increasing energy supply resilience. We have also described a case study that reflects on the potential impacts of FPVs in Brazil, with the details of calculations available in the supporting information. This case study is an example of FPVs potentials in developing countries. The provided details help the readers with interest in calculating FPVs potential for other locations to use this approach and determine the FPVs electricity generation for the desired areas. The last sections of this article, challenges for FPV implementation and conclusion, consider opportunities as well as barriers that despite this technology significant potential may hinder the market expansion. This transdisciplinary article aims to offer a new perspective and provide a bigger picture of this emerging technology hoping to stimulate FPVs related discussions and initiate new studies.

**Figure 1:**
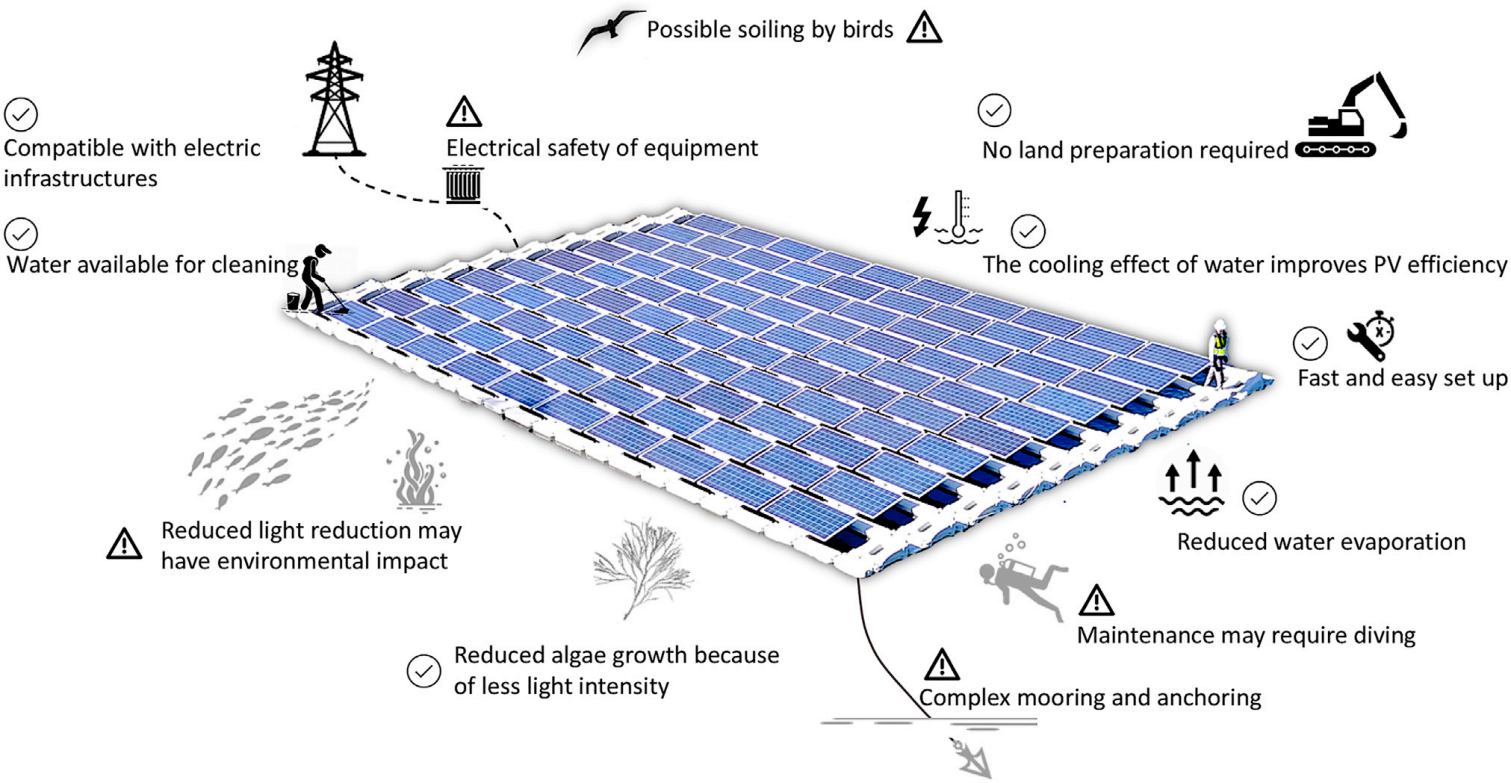
Schematic representation of some of the main advantages and potential disadvantages of the FPV systems

The idea behind FPVs is simple; an array or combined arrays of PV panels are placed on floating structures that keep them above the water surface (Spencer et al., 2019). Such floating infrastructures are susceptible to a range of environmental risks that could jeopardize the long-term performance of these solar farms. Fluctuations in water levels, heavy storms, earthquakes, and tsunamis are some of these potential risks. Even in light of such concerns, the strong performance and reliability of the existing floating solar farms have convinced many countries to express interest in this system (Pouran, 2018a, 2018b). Figure 1 illustrates the benefits and risks of this technology. A growing number of companies have already started joining the floating solar market either by providing different designs of FPVs like rotating/tracking systems or offering other customized products designed for floating solar power plants. An example of the latter one is the PV panels tailored to minimize the corrosion of the module when using over water, with stronger wet-proof properties and lead-free ribbon to prevent water pollution.

The megawatt-scale FPVs emerged from a 1.1-MW floating power plant built on a rainwater retention pond in Okegawa city in Japan in 2013 (Pouran, 2018a, 2018b). The second milestone was the 6 MW project on Queen Elizabeth the Second reservoir near London (completed in 2016) (Lightsource bp, 2019); however, the market was not paying enough attention to this technology, until China changed the game. The inauguration of the world’s largest floating solar power plant, 70 MW, on a collapsed coal mine in 2017 raised hopes about the commercial viability of FPVs. Since then, FPVs have experienced significant growth in scale, geography, and design. A recently completed 17-MW FPVs in south of France (Akuo, 2022), high-altitude FPV at height of 1,810 m in Switzerland that could produce 800,000 kWh of electricity per year (House of Switzerland, 2021), and Ocean Sun technology that comprises modified PV modules attached to a thin, flexible floating membranes are prime examples (Ocean Sun, 2022). A recent study shows that high-altitude FPVs on Swiss water bodies could provide the total national electricity demand for this country, which reflects the electricity generation potential of this technology even in geographically challenging locations (Eyring and Kittner, 2022).

A study by the US National Renewable Energy Lab (NREL) suggests that FPVs can generate around 10% of the US annual electricity production. This estimate is based on deploying this technology on man-made reservoirs that are not facing any legal or environmental constraints, which amounts to over 24,000 water bodies (Spencer et al., 2019).

**Figure 2:**
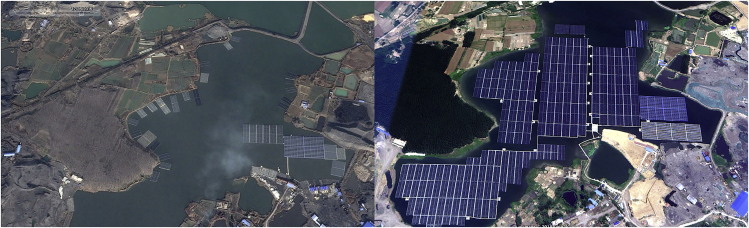
Early and late stages of installing 70-MW FPV on a flooded coal mine in Anhui, China (Credit Google Earth)

A challenging aspect of floating solar power plants is their building costs, which are higher compared with their ground-mounted peers. No land preparation is required; however, the location of the water reservoirs and the logistics affect the financial requirements of these systems. Characteristics of the water body, for example, depth, water level variations, soil/bedrock, and the type of floats used to support the PV modules also influence the deployment costs of FPVs. Although the number of companies involved in FPV technologies is growing and they offer different innovative designs, we could have the following cost estimate based on the technology used in the Anhui project. For a multi-MW large-scale project, if the floats are manufactured locally, waterbody characteristics do not cause anchoring complications, and the local labor force is used, achieving close to 1 USD/Wp for installing FPVs is realistic. (Pouran, 2018a, 2018b). The long-term performance of the FPV system, which is often designed to stay in place for as long as 25 years, to a large extent relies on the stability of anchoring of these power plants. Anchoring also constitutes an important part of the expenses. The reported anchoring costs for the Anhui project in China, Figure 2, are around 10 USD/kW, which is shallow water, is a large-scale project, and has benefited from the local manufacturing facilities and labor force. But in Japan, the anchoring price is substantially higher, and the aim is to reach 30 USD/kW or less (Pouran, 2018a, 2018b).

The latest figures regarding global deployments of FPVs belong to the International energy agency (IEA) Photovoltaics Power Systems Programme (PVPS) report that suggests by 2021 the installed capacity of FPVs has surpassed 3 GWp globally, which is more than 20% of what has been achieved in 2020 alone (Masson and Kaizuka, 2022). In research conducted by the Word Bank, ESMAP and SERIS estimate that, even if 1% of the total surface area of man-made reservoirs is covered by floating solar, the global FPV capacity would reach 400 GW (World Bank Group et al., 2018). This excludes the potential of offshore FPVs, which countries like the Netherlands have already started considering as part of their energy portfolio (Greentech Media, 2020; Reuters, 2018). In principle, like all other energy generation methods, the environmental impacts of FPVs, similar to ground-mounted solar, are not negligible as the manufacturing process of PV modules, inverters, floating parts, and all other components of such systems involve energy consumption and greenhouse gases emissions. Nevertheless, the positive impacts of floating solar technology are far beyond generating clean, green, and renewable energy. They provide other social, technical, and environmental benefits that improve sustainable and economic development of societies and facilitate achieving energy trilemma (affordability and access, energy security and resilience, and environmental sustainability [Jing et al., 2021]). So far, the development of FPVs has been mainly focused on onshore deployments, considering the harsh marine environment could jeopardize the long-term stability of such structures. Typhoon Faxai that hit Japan in 2019 and caused fire in one of the floating solar power plants, while rare, still highlights a real risk that could discourage investment in this technology (Bellini, 2019).

### Socio-environmental impacts of floating solar farms

The long-term success of emerging low-carbon technologies, including FPVs, requires public support and attention to the socio-environmental impacts, which is a complex and interdisciplinary topic by nature. Recently we have seen an increasing number of studies on technical, financial, and to some extent environmental aspects of FPVs as an emerging system. yet only limited information is available on their social aspects. As Bax et al. (2022) suggest, negligible or no published research exists in which the social or societal implications of FPVs are being examined. We acknowledge that a strong social study would consider the application of interviews with impacted communities to understand their perspectives like a recent and rare study of exploring the social feasibility of floating solar energy infrastructure in the Netherlands (Bax et al., 2022). However, such studies are beyond the scope of this article and here we briefly reflect of on three key socio-environmental impacts of FPVs: job creation, non-occupation of habitable land, and improving water security in water-scarce regions.

### Jobs creation

An important socio-economic impact of the FPV system installation is the potential of job creation. The solar industry creates both direct and indirect jobs including PV and float manufacturing, installation, maintenance, retail services, electrical devices, and public officers. (Pouran, 2018a, 2018b; Sooriyaarachchi et al., 2015). The role of PV solar in creating jobs varies significantly across countries and is influenced by different factors including the labor market. For example, it is estimated that in Turkey each MWp of PV system can create up to 395 jobs, which includes installation, operation and maintenance, panel production, and retail services. From this number three jobs belong to maintenance and operation for 1 MWp of PV (Çetin and Eĝrican, 2011). On the other hand, a study by the International Labour Office (ILO) suggests that PV solar creates about 6 jobs per MW of capacity in manufacturing, construction, and installation and 1.2 to 4.8 in operating and maintenance (ILO, 2011), which means 7 to 11 full-time employments. The estimated total number of jobs created by the PV solar industry varies to a notable extent (Jenniches, 2018); for instance, some studies suggest a minimum of 12 and a maximum of 70 are created per MWp (Cameron and Van Der Zwaan, 2015). This information and estimates belong to ground-mounted PV solar, which might present a slightly different job landscape compared with FPV. Even it is expected that we have different numbers of job opportunities created by offshore and onshore FPVs. However, those employed by the PV solar and hydropower sectors can provide significant overlapping and transferrable skills to the FPV power plants.

A World Bank report indicates that, even under conservative estimates, the global potential of FPVs is more than 400 GW (World Bank Group et al., 2018). While acknowledging the uncertainties in the number of jobs created by the PV industry, and the differences between PV and FPV projects, if we consider a minimum (ILO, 2011) of 7 and maximum (Cameron and Van Der Zwaan, 2015) of 70 full-time jobs per MWp, the floating solar market of 400 GW could create between 2,800,000 and 28,000,000 employment opportunities. The designing stage of FPVs and installing anchors are considerably more challenging than designing and installing ground-mounted solar. This is because they need complex anchoring systems that allow these islands to withstand various changes in environmental conditions, including the fluctuation of water levels in the reservoirs over the years. (Pouran, 2018a, 2018b) The design of an FPV depends on many factors including the water depth, the properties of the waterbed, wind velocity, and annual precipitation. Suppose such an FPV system is designed for a low-depth lake and does not require heavy machinery and equipment, e.g., for anchoring and moving different types of buoyant structures. In that case, the number of employed people during the installation step is less than the ground-mounted PV as no land preparation is required. Maintenance, including cleaning the PV panel surfaces, is another difference between PV and FPV systems. For the FPV, sitting on the water makes the panels less susceptible to being covered by dust and other particulate matter (Zahedi et al., 2021). Nevertheless, their maintenance may still require the same or more personnel than the PV farms as the buoyant devices and devices that are in constant contact with water need to be regularly monitored. It is worth noting that the demand is higher for smaller-scale PV projects (from 1 to 5 MW capacity) than for large-scale ones. This scale of solar farms is creating more job opportunities in this sector (Pimentel Da Silva and Branco, 2018). We expect this to be the case for the FPVs as well because of the nature of FPVs and the scales of installed FPVs so far (Pouran, 2018a, 2018b). In countries like India, when domestic production, testing, and product design are possible, FPVs can potentially create more jobs (Manoj Kumar et al., 2022).

The promise of FPV technology lies in its flexibility and adaptability with different water bodies (World Bank Group et al., 2018). It can also be used in brownfields, which are areas that have been affected by the former uses of the site and have real or perceived contamination problems (Hammond et al., 2021). Brownfields can be considered as environmental liabilities and require intervention to bring them back to beneficial use; a prime example is coal mine areas. Brownfields that can form permanent or seasonal reservoirs are well positioned for floating solar farms. They are often located near electricity transport and infrastructure, which would reduce the costs of building these systems. If the brownfields, which are often overexploited and abandoned, need to be redeveloped, the FPVs can be dismantled and used in other locations or recycled. These power plants also need maintenance personnel, which local people can train. The provision of energy and job that stemmed from adding FPVs to abandoned brownfields would benefit the local communities and create a safer and healthier environment. The Anhui flooded coal mine project is an excellent example of such benefits. Instead of migrating looking for jobs, local people who have been working underground some years ago now are the maintenance personnel of this solar farm (Pouran, 2018a, 2018b). It is worth noting that, even for the above example, Anuhi FPV project, the environmental liability is still there and can potentially cause environmental impacts. This indicates that, although FPVs could be an important part of the solution, they cannot address all the environmental concerns associated with a brownfield site.

### Non-occupation of habitable or productive areas

The versatility of these systems allows deploying them even on drinking water reservoirs reducing the conflicts over land, which is particularly important in countries like Japan, where the appropriate areas for ground-mounted solar farms are too expensive or, if available, they are mostly not flat, which is a concern to ensuring the safety of PV systems. For instance, some studies show that land available for PV and wind turbine development accounts for 0.9% of contiguous land in Japan. Of the available land, 72% requires competition between PV and onshore wind systems (Obane et al., 2020).

Another example is Vietnam, where the annual growth rate of electricity demand has been 12% in recent years, faster than any other comparable Asian economy (Dapice et al., 2018). To maintain economic growth, Vietnam needs access to a secure energy supply. Increasing coal imports so far has been the main way of Vietnam’s government’s response to the growing energy demand (which is an unsustainable approach). On the other hand, close to 40% of the total land area in this country is dedicated to agricultural production, which generates 15% of the gross domestic product but provides employment for 40% of the population (FAO, 2018). Floating solar farms provide a unique opportunity for Vietnam to address its growing energy demands and transition to a low carbon economy by utilizing a small portion of thousands of hectares of water surfaces available for this purpose. Deploying FPVs would mitigate land-use conflicts; generate a clean, secure, and reliable source of energy; increase efficiencies of agriculture and aquaculture, as well as improve the welfare of those employed in these sectors (Pouran et al., 2021) The benefit of mitigating land-use conflicts for renewables through deploying FPVs is not limited to the above-mentioned countries, and the number of nations relying on this technology is growing, for example, Thailand, Singapore, and the Netherlands.

FPVs could also potentially address the concerns related to the impacts of renewable energy development on biodiversity. Land-use intensive infrastructures, especially solar PV, occupy large areas of land by panels, which, if not planned correctly, can lead to habitat conversion or habitat loss resulting in impacts beyond the immediate physical footprint (Rehbein et al., 2020). Despite such benefits, public perception and acceptance of FPVs is a topic that might hinder the expansion of this technology to different types of water bodies, a topic that is currently largely unexplored. In the first published study that involves interviewing different stakeholders, Bax et al. (2022) evaluated public support for the deployment of an FPV pilot project at a recreational lake in the Netherlands. This research showed that the diversity of stakeholders and their diverging use of the lake leads to a variety of concerns about how the pilot project could affect their activities and interests. The results highlighted that the stakeholders with high dependency on the lake, e.g., recreational assets, consider the pilot project to pose a significant threat to place-based values and activities. The existing uncertainties on possible impacts due to the newness of FPV have been identified as the major reason for stakeholders’ reluctance toward this pilot FPV. As the above study suggests and as mentioned earlier, FPV is an emerging technology. Its social impacts are expected to vary based on the scale, nature of the waterbody, local population, and project-specific conditions. In such context it is understandable to see drastic difference between the supportive views of unemployed locals who had found employment opportunities when FPVs were installed on the abandoned coal mine in China (Pouran, 2018a, 2018b) and doubtful views of the impacted population of recreational lake in Netherlands (Bax et al., 2022).

It is worth noting that, while we see a significant increase in numbers and geographical distributions of FPV deployed on inland water bodies, offshore FPVs currently have a very limited deployment. This can be attributed to the harsh sea conditions. Nevertheless, the possibility of using offshore FPVs including hybrid offshore winds and solar is getting traction (Lopes et al., 2020, 2022; Soukissian et al., 2021). It is a key development as non-occupation of productive areas is particularly crucial for many islands and countries that extensively rely on fossil fuels and land scarcity prevents sufficient deployment of conventional renewables. A recent study focused on the Maldives as the archipelagic country has shown the crucial role that offshore floating technologies can play in the energy transition of islands from fossil fuels to clean energy (Keiner et al., 2022).

### Improving water security in water-scarce areas by reducing the evaporation

One of the benefits of FPVs compared with the common PV technology is its role in reducing water evaporation from the water bodies that they cover. A study by Lopes et al. (2020) on the Brazilian regions with semiarid climate showed that, with FPVs coverage scenarios of 20%, 50%, and 70%, we could expect 15.3%, 37%, and 55.2% in water evaporations reduction, respectively, from the reservoirs. This is crucial for increasing the resilience of cities in such climates, particularly during drought times. Experimental studies conducted in arid and semiarid regions worldwide have shown that covering the water surface with FPV can effectively reduce the evaporation of reservoirs (Lopes et al., 2020). Unlike Japan, many countries, for example, those in the Middle East and North Africa (MENA), have no difficulties in allocating lands for PV solar farms; still, they are keen on FPVs and looking at this technology from a different perspective, to mitigate water evaporation (Asaba, 2019). Water scarcity is a severe problem in the MENA region, a challenge that would become more pressing as climate change brings prolonged droughts and heatwaves and accelerates evaporation from open water reservoirs (Gosling and Arnell, 2016). Studying the impacts of FPVs on mitigating water evaporation is quite new; nevertheless, a growing number of researches suggest this impact is notable (Lopes et al., 2022). This feature allows countries in the arid regions to incorporate more renewables into their energy mix, reduce greenhouse gas emissions, and decrease water evaporation. The air temperature, heat flux, mass transfer in the water surface, and wind speed are the most critical factors determining the reservoir’s evaporation rate (Fereshtehpour et al., 2021). The extent of the evaporation reduction is directly affected by the FPV design, e.g., the shape and size of the floats that support the PV modules and island as well as the ratio of the covered surface to the reservoir surface area (Pouran, 2018a, 2018b). The energy generated by these systems could also be used for the desalination of seawater in coastal areas and improve access to clean water and sanitation, particularly in the MENA, where desalination is crucial for providing drinking water (Pouran and Hakimian, 2019).

PVs have been installed on the top of water canals in another approach to reduce water evaporation in arid climates. They are not entirely water based and are mounted on the ground. The 10-MWp utility-scale grid-connected canal-top PV power plant in India is a prime example (Manoj Kumar et al., 2022). Interestingly, field studies showed that the performance of this PV plant is slightly lower than land-based plants, which is attributed to the high humidity of the PV panels’ ambient condition (Kumar et al., 2020). However, this could vary under different climates, and the cooling effect of water has been shown to be an important factor in improving FPV panels' performance (Lopes et al., 2022). Mitigating water evaporation is a key advantage of this type of installation with a significant contribution to sustaining energy-water-food nexus. A study that simulated the benefits of canal-top PV installations in California shows that such a system could reduce annual evaporation by an average of 39 ± 12 thousand cubic meter per km of canal (McKuin et al., 2021).

## Technical benefits of FPVs

### Use of degraded areas

Adding PV solar farms require extensive land preparation that could add to deforestation, habitat conversion, and loss of fauna and flora (Choi, 2014). On the other hand, in many regions across the world, we have brownfields that once have supported economic and industrial activities and are now abandoned and underutilized. They are often contaminated and pose an environmental and human health risk, and their remediation is costly and time-consuming (Pouran et al., 2018). Brownfields are also frequently located close to settlements, provide the opportunity for further development, and reduce environmental and resource deterioration, the extension of infrastructures, and transportation costs (Ameller et al., 2020).

In this context, FPVs can be deployed on brownfields, even the contaminated ones without the need for bioremediation, for short or long term. A prime example is the extensive deployment of FPVs on contaminated lakes of the collapsed coal mines in Anhui Province in China (Pouran, 2018a, 2018b) where local people who used to work as miners are working in jobs related to the FPV, assembling solar panels, and at the maintenance. Consequently, they have exposed less harmful work conditions. A study on a mine pit lake in Korea (Song and Choi, 2016) suggests that FPV deployment on the pit lake of the abandoned mine is economically feasible, with an annual reduction of greenhouse gas emissions of twice the reduction effect that can be achieved by reforestation of the abandoned mine site. The study concludes that FPVs can be considered as an efficient approach for reusing brownfields, including abandoned mines (Song and Choi, 2016), a conclusion that has been reiterated in other studies as well (Pouran, 2018a, 2018b; World Bank Group et al., 2018).

Solar PV site suitability studies consider land slope and morphology as some of the most important criteria for site selection for PV projects (Al Garni and Awasthi, 2018). They are not only important for the long-term reliability of these infrastructures but are also crucial for the optimized design of PV farms to prevent partial shading that plays a vital role in the reduction of the output power of these farms (Tatabhatla et al., 2019). Unlike the land surface preparation requirements for solar PV, for FPVs the water surface is perfectly flat, does not require preparation, and reduces the effect of mutual shading of the rows of the modules. Land occupation is limited to the energy transformer substations and cable ducts, also the visual impacts of FPVs compared with the more common PVs are significantly less as they sit on the water surface.

### Reducing algal bloom in reservoirs

When deployed on water reservoirs FPVs can have a positive impact on reducing the rapid increase in the population of algae known as algal bloom. Algal bloom can pose serious negative impacts on water supplies, fisheries, tourism, and recreational uses of water bodies (Paerl et al., 2018). The presence of FPVs reduces the sunlight penetration underwater, as well as the water temperature. Sunlight is crucial for algal growth and is required for photosynthesis, and the shading provided by the FPV can mitigate algal proliferation and improve the water quality (Sahu et al., 2016). This is particularly important for the freshwater bodies as excessive algal growth could significantly reduce water quality (Cazzaniga et al., 2018). Lower algal growth also reduces the likelihood of developing toxic species and the release of their toxins (Haas et al., 2020). The extent of the impact of FPVs on mitigating algal bloom depends on the characteristics of the water bodies, including nutrient availability. Nevertheless, FPVs limit the photosynthesis of algae, which is critical for their expansion.

In a study that modeled the impacts of FPVs on algal bloom, considering chlorophyll-a as a proxy for biomass and microalgal growth, Haas et al. (2020) showed that, for the water surface coverage below 40%, the modules have little or no effect on microalgal growth, whereas 40%–60% coverage could cause a notable decrease in algal blooms because of significant reduction of light in the reservoir. The study suggests that a more extensive deployment of FPVs could potentially eradicate algal blooms, which could affect the entire water body ecosystem. Their research has considered Rapel hydropower reservoir in Chile as it frequently experiences toxic cyanobacterial growth. It is expected that the nature of the water body, its location, and the ambient environment, as well as its function, type, and design of FPVs, affect such impacts. Understanding how FPVs and their various designs and deployments could change the extent of algal bloom require extensive studies and comprehensive field analysis. Nevertheless, it is highly likely that reducing solar light penetration underwater by FPVs reduces algal growth. A recent field research in Mahoni Lake in Indonesia relying on mesocosm experiments to evaluate the impacts of FPV on the lake ecosystem has demonstrated that water chlorophyll-a concentration is reduced if we have extensive FPV coverage of the water surface (Suwartha et al., 2022).

### Increasing the PV panel efficiency because of reduced ambient temperature

In a PV panel absorbed solar energy in addition to generating electricity generates heat that raises the temperature of the PV cells. The increased temperature of the solar panel cells reduces the efficiency of the panel and, therefore, the energy output. Previous studies have shown that reducing the PV panel temperature and ambient heat would improve the energy outputs; examples include designing cooling ducts to minimize efficiency loss (Brinkworth and Sandberg, 2006) or even experiments on submerged PV panels (Rosa-Clot et al., 2010).

Efficiency loss because of the high ambient and operating temperature of the PV panel depends on the technology used. Changes in the efficiency per one degree Celsius changes in the PV cell temperature are shown in %/°C; it could reach about −0.45% per °C for commercial PV panels (Cazzaniga et al., 2018). In general, the nominal power of a PV panel is the output measured at 25°C and any temperature above that will reduce the output efficiency.

In FPVs the PV modules' operating temperatures tend to be lower due to the evaporative cooling effect of the water, which improves the panel efficiency. If aluminum frames are used for supporting the floating solar PV modules, they carry the cooler temperature from the water, reducing the overall temperature of the module (Sahu et al., 2016). Some studies suggest that on average the efficiency of FPVs is about 11% more than the ground-mounted solar panels (Choi, 2014). The impact of the cooling effect of the water on the PV panel depends on the FPV design and float structures as well as ambient conditions, e.g., wind speed; nevertheless, regardless of the design, the FPVs have a lower temperature compared with the common solar farms (Kamuyu et al., 2018; Suh et al., 2019). A recent study in Indonesia on FPVs based on remote sensing analysis has shown that there is an 8°C difference between the surface temperature of a lake and its surrounding environment (annual average) (Sukarso and Kim, 2020).

As mentioned before, FPVs, because of their locations, are also less susceptible to dust deposition, which means on average they receive more sunlight compared with their peer ground-mounted solar farms. The presence of dust particles in the atmosphere not only covers the panel surface but also scatters solar irradiation reducing the efficiency of a solar farm.

### Complementing the existing hydropower infrastructures

According to the International Energy Agency (IEA) hydropower is expected to remain the world’s largest source of renewable electricity and keep playing a crucial role in decarbonizing the energy system with 1,307 GW installed global capacity (by 2019) (IEA, 2020). It is estimated that hydropower has produced about 16% of the total global electricity in 2018 (4,210 TWh of the total 26,700 TWh) (C2ES, 2021). The hydropower potential is affected by the hydrological regime, which is a function of regional climate patterns and would vary as climate changes (Chilkoti et al., 2017). Changes in precipitation, evaporation, and runoff, because of climate change, affect the variability and volume of streamflow. The projected impacts of climate change suggest an overall decrease in hydropower potential (Yalew et al., 2020). For arid and semiarid climates, the impact of climate change would be more severe and the increased annual average temperature in addition to prolonged, more frequent, and severe heatwaves and droughts would put more stress on available water resources and accelerate evaporation from the dam reservoirs leading to reduced hydropower potential (Fereshtehpour et al., 2021). A prime example is the water level at Lake Powell, on Colorado River, which in March 2022 fell to its lowest threshold since the lake was created by the damming of Colorado in 1963. This water shortage challenge is attributed to extensive droughts in the western United States, which also threatens energy production by the Glen Canyon hydropower dam. This concern can be exacerbated in the long term as prolonged droughts, heatwaves, and evaporation will reduce the water behind the dam to levels below the minimum water at which turbines can produce hydroelectric power (Bittle, 2022).

FPVs are compatible with different water bodies including hydropower dams reservoirs. Retrofitting hydroelectric dams with FPVs would increase the total output of these systems by adding efficient and clean solar energy, which also benefits from existing grid connections and maintenance personnel. As mentioned before, the PV panels on the water surface also benefit from the cooling effect of water, reducing the system’s operating temperature, preventing overheating of the solar panels, and improving the energy yield (Kamuyu et al., 2018; Suh et al., 2019).

**Figure 3:**
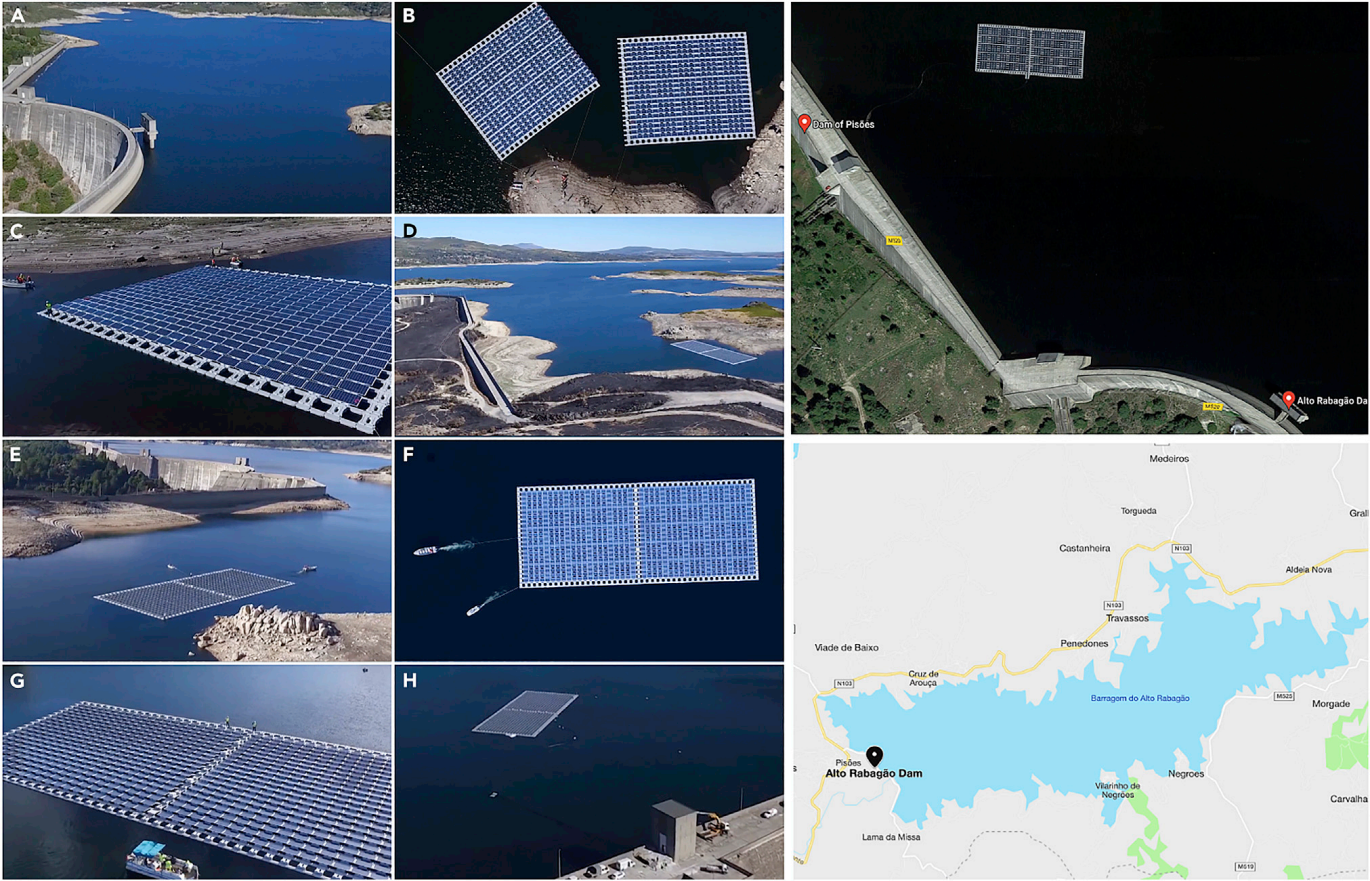
Integration of FPVs with a hydroelectric power plant in Portugal (A–H) Assembly stages and installation of the 220-kW FPV on Rabagão dam reservoir in Portugal (Credit EDP Energias de Portugal). The top and bottom images on the right show satellite images of the installed FPV and the location of the dam, respectively (Credit Maps by Apple).

Such integration would increase the capacity of these dams to meet the energy needs during peak hours, particularly in dry seasons. Seasonal variability of water levels behind the dams is significant, particularly in the arid and semiarid regions, a trend that would be accelerated because of the climate change effects and prolonged heat and drought. The presence of FPVs would reduce water evaporation from these reservoirs (Fereshtehpour et al., 2021). It is worth adding that, particularly compared with the arid regions, water reservoirs provide a clean ambient environment, which reduces the presence of dust settled on the panels and, therefore, maintenance costs. The world’s first combined FPV and hydroelectric system were grid-connected in 2016 as an installation of 220 kW FPV on Portugal’s Rabagão Dam (Climate Action, 2017; Farfan and Breyer, 2018). Figure 3 (EDP Energias de Portugal, 2018) shows the potential of this hybrid system. A study on integrating FPVs in hydropower dams in Brazil shows that these systems could enhance the existing power sources and be considered as an alternative approach toward meeting the energy demand growth without building new dams (Sulaeman et al., 2021).

It has been also suggested that adding FPVs to hydropower systems would significantly improve the overall system reliability, minimize load curtailment, and provide more flexibility to the operator during peak times. For African hydropower, the capacity of adding FPV to the dams’ reservoirs is estimated to be more than 2,900 GWp. Even only 1% of FPV coverage doubles the installed capacity and increases electricity output by about 58% (in addition to saving more than 740 mcm/year of water) (Gonzalez Sanchez et al., 2021). Supplying the electricity demand with solar energy during peak irradiation hours and balancing grids with hydropower during low and no irradiation times are of unique potentials of FPV integrated hydropower systems (in addition to other benefits including relying on existing grid facilities, saving water, and no need for land preparation) (Farfan and Breyer, 2018; Zhou et al., 2020). Determining global potential FPV hybridized with hydropower intrinsically involves notable uncertainty; nevertheless, it is significant and is estimated to be between 3.0 and 7.6 TW (Lee et al., 2020).

### Electricity supply decentralization and infrastructure resilience

FPVs will contribute to the decentralization of the electricity supply, adding new resources to the system (Pouran, 2018a, 2018b). This diversification and decentralization make the infrastructure more resilient (Kang et al., 2020) especially as climate change impacts will intensify the frequency and severity of hurricanes, heavy storms, heatwaves, and droughts. In countries like those in Southeast Asia that have a high number of wetlands and permanent and seasonal water bodies scattered across their land, small-scale FPVs can be deployed extensively throughout the country, e.g., on water bodies that are used for agriculture. This would also empower the smallholders as Prosumers (consumers producing electricity) (Palm et al., 2018). Small-scale FPVs could improve the electricity sector by bridging consumption and generation. They would also have profound impacts on the market and facilitate public participation in investment in sustainable infrastructures and capacity building as well as making the entire energy supply more resilient through supply decentralization.

The expensive electricity can reduce the competition of some products in the market and hamper the commercialization and the feasibility of enterprises. The reduction in the cost of energy and the feasibility of production processes was the reason for installing the first commercial FPV plant in South Africa, Western Cape (Caboz, 2019). The FPV of 60 kWp is used to supply the fruit farm with irrigation pumps, illumination, packaging, and cold storage of fruits (Caboz, 2019; ESI Africa, 2019).

The unique potential of FPVs has led to conceptualizing different possibilities that this technology might provide in diversifying energy supplies. A notable example is the concept of solar methanol islands, which uses solar energy from large-scale FPVs islands to recycle atmospheric carbon dioxide into methanol. As the authors of this article have acknowledged, this theoretical work aims to initiate questions in related scientific disciplines that could lead to the possibility of the development of such applications for FPVs (Patterson et al., 2019). Nevertheless, such studies (Keiner et al., 2022; Patterson et al., 2019) reflect the opportunities that combining FPVs with the existing technologies could provide.

### Potential negative environmental impacts of floating photovoltaics

Negligible information is available on the environmental impacts of FPVs (Lopes et al., 2022; Pouran, 2018a, 2018b). However, some negative implications can be envisaged if the FPVs are deployed in small water bodies particularly closed lakes and engineered pools that are used for agriculture and aquaculture. These potential impacts are inferred from other industries, as such studies have not been performed for FPVs yet. These negative environmental impacts and the conditions that could cause them include:

• If the design of the FPV and floats restricts oxygen and gas exchanges between the water surface and ambient environment it could lead to anerobic condition, which affects microbial community and water chemistry of the ambient ecosystem (Oswald et al., 2015; Pouran et al., 2018; Roland et al., 2018).
• When the solar radiation that penetrates the water is substantially reduced, due to coverage of most of the water surface by PV panels, the water ecosystem might be affected negatively (Haas et al., 2020). This is despite the positive impact on algal growth reduction. Water temperature also will deviate from natural levels, which is caused by coverage of the surface by PV panels and floats.
• Risks of chemical pollution because of sudden or gradual releases of chemicals during the installation, maintenance, or lifetime of the FPVs are also of the potential concerns. This could include chemicals that compose PV panels or are part of the floats or electrical and mechanical equipment of the FPVs components.
• Oil and fuel leakage/spillage when they are used directly or indirectly for the installation and maintenance of FPVs, e.g., from boats that are used for moving the floats to the planned location within the water body.
• Corrosion and degradation overtime could release chemicals and microplastics (Kirchgeorg et al., 2018; Pouran, 2018a, 2018b) from different components used in FPVs.
• Electrical equipment, especially those that are directly in contact with water, could create electric fields, which might have an impact on the environment.
• Underwater noise pollution during the construction of FPVs (Vakili et al., 2021).
• Fire incidents, using fire extinguishers and sink of the FPVs floats could cause chemicals release into the water ecosystem.

The above, although some of them are unlikely, nevertheless, indicate the environmental risks that could be attributed to FPVs and need further research.

## Initiatives in developing countries

Considering the demonstrated benefits, such as reduced evaporation, inhibition of algal growth, and greater efficiency of PV generation, FPV is likely to be an attractive option for many countries. Furthermore, this technology could contribute to economic growth in developing countries by promoting access to clean energy and developing local industry and infrastructure. The energy generated by the FPVs could be used for multiple purposes, such as water desalination systems, pumping water for irrigation and farming, or residential consumption.

India, for example, has presented a remarkable deployment of renewable energy installed capacity, growing almost 3.5-fold in recent years, mainly from wind and solar technologies (Acharya and Devraj, 2019). FPVs are in an early stage of development in this country, but its capacity is increasing rapidly. According to Acharya and Devraj (2019), over 1.7-GW capacity projects were reported in 2019 at various stages of development. The first project was a 10-kW FPV plant on a pond in Rajarhat in 2015, sponsored by the Ministry of New and Renewable Energy. In 2016, in Kerala, a 500-kW plant at Banasura Sagar reservoir started the operation, and in 2018 a 2-MW project was commissioned at Visakhapatnam, Andhra Pradesh (Acharya and Devraj, 2019). Recently, Bharat Heavy Electricals Limited has commissioned India’s largest floating solar PV plant, with a power capacity of 25 MW and an area of 100 acres. Located at Simhadri thermal station in Andhra Pradesh, it aims to produce clean power, reduce water evaporation, and save valuable land resources (Punj, 2021).

India has a large number of man-made reservoirs, which are used for a variety of purposes like hydroelectric, water supply, and irrigation (Acharya and Devraj, 2019). Furthermore, the Energy and Resources Institute has undertaken a study to analyze the potential of FPV in the country. About 18,000 km^2^ of water surface area is suitable for installing FPV plants, with an overall potential of 280 GW (Acharya and Devraj, 2019).

Brazil is another example. This country presents considerable FPVs installation potential due to the availability of water and solar resources (Lopes et al., 2022). According to Lopes et al. (2022), if FPV systems cover only 1% of the identified suitable areas, they can produce 79,377 GWh/year, equivalent to almost 12.5% of the current national generation and 16% of all the country’s electricity consumption. In this context, hydropower plants stand out, representing approximately 72% of the installed capacity potential.

With an electrical matrix highly dependent on hydroelectric generation, water scarcity directly impacts the country’s electricity supply. However, the installation of floating solar plants in the country is still in the early stages. The largest FPV project is in the reservoir of Sobradinho Hydropower Plant in Bahia (EPE, 2021a). The first stage of the project has a capacity of 2.5 MWp (EPE, 2020). Also, a 5-MW FPV plant is installed at the Balbina hydroelectric plant in Amazonas. The structure was created to increase local energy production since the drought significantly reduced the generation of hydro power plant (HPP) (EPE, 2020).

### Impacts evaluation: A case study in Brazil

Hydroelectric plants represent more than 62% of the installed capacity of the National Interconnected System (SIN) and are the main source of energy storage in Brazil (EPE, 2021b). Droughts, heatwaves, and reduced precipitations because of climate change can significantly reduce the water levels of the hydroelectric dam, which jeopardies energy production. Hydropower integrated FPVs could mitigate this challenge by co-producing clean energy and reducing water evaporation that helps the water scarcity as well (da Costa and da Silva, 2021).

To demonstrate the crucial role that FPVs could play if they are integrated with hydropower here, we have calculated the potential of FPVs deployment on Balbina hydroelectric dam, in Brazil. It was built during 1980s to supply Manaus city, in the Amazonas state of Brazil, replacing and complementing the local thermal generation parks. For this purpose, an area of 2,360 km^2^ of the Amazon rainforest was flooded, which involved the expulsion of the local people and indigenous Indian communities. Balbina hydropower has five turbines of 50 MW and a total capacity of 250 W installed. However, the electricity generation history from 2013 to 2021 shows that the average production has been 115 MW, with an average minimum of 55 MW in 2016 and a mean maximum of 164 MW in 2021 (ONS, 2022). In other words, the Balbina plant has worked with an average capacity factor of 46% in this period, which is comparable with the capacity factor of other Brazilian hydro plants for the same period, 42.4%, based on ONS (2022) and CCEE (2022) data. This information reflects a critical point and highlights that just half of the transmission system capacity is used. FPVs can mitigate this issue and increase the use of transmission lines, substations, and flooded areas of hydroelectric reservoirs. Based on this concept, we have evaluated the potential of floating solar deployment and have calculated how much the power generation could be improved if we have FPV integrated at the Balbina reservoir. The details of our calculations and technical information including the software we used for this purpose, System Advisor Model (SAM), are provided in the supporting information. These details are helpful for reproducing this approach and calculating the FPV potentials for the desired locations.

The calculated results are shown in Table 1. One of the most striking aspects of these results is the energy generation based on the occupied surface area. The occupied area by 1 MW of FPV system is 10,512 m^2^ (Padilha Campos Lopes et al., 2020). The calculation of occupied area of Balbina hydropower considered the average generation since 2013 to 2021 divided by the area of reservoir. The capacity factor for the FPV plant in this study is determined by the software SAM. It corresponds to the average energy output divided by the output if it had operated at full (rated) capacity over the same period.

**Table 1:** Comparison between FPV system and Balbina Hydropower

Electricity generation technology	Average annual energy generation (MWh)/1 MW installed	Annual Energy generated/area occupied	Capacity factor
FPV plant	1,408	133.9 kWh/m^2^	16.1%
Balbina Hydropower	4,048	0.37 kWh/m^2^	46.2%

As seen in Table 1, Balbina hydropower needs more than 360 times of flooded area, reservoir surface, to produce the same quantity of energy compared with the FPV plant. It is worth noting that the needed reservoir surface to generate certain amount of hydropower to a great extent depends on the topography of the reservoir. However, in this case, to produce an average energy of 1,012 GWh by Balbina hydropower installation of 719 MW of FPV is required. Such capacity is about 5 times higher than the current world’s largest FPV plant based in Anhui, China, with a capacity of 150 MW, and more significant than the proposed 600-MW FPV plant project in India that will gradually grid connected in the coming years (Nassa et al., 2021).

Nevertheless, the integration of FPV could still optimize electricity production through the Balbina hydropower infrastructures. If 86 MW of FPVs is deployed in Balbina, it would optimize the use of the existing substation and the transmission lines. The 86 MW of FPV could produce 119,680 GWh per year, covering only 0.03% of the Balbina’s reservoir. Such added capacity would improve the average capacity factor from 46% to more than 64% (please see supporting information for further information). Another implication of such a hybrid system is creating new job opportunities. If we consider a minimum of 7 (ILO, 2011) and a maximum of 70 full-time jobs per MWp (Cameron and Van Der Zwaan, 2015), the installation of this 86-MW FPV plant could generate between 600 and 6,000 full-time jobs.

## Challenges for FPV implementation

Deploying FPVs in different locations, from high-altitude FPVs in Switzerland to brownfields in China and a hydroelectric dam in Portugal, demonstrates the versatility of this technological concept growing in various designs and sizes and applications. Defining the expected impacts and the criteria to achieve them are important factors that affect the expansion of FPVs installations. For example, while proximity to transmission lines and the area, type, and size of a waterbody could be readily quantified as engineering criteria, defining environmental and social dimensions of deploying an FPV project involves significantly more complex factors. The novelty of this technology and uncertainties related to such impacts, particularly when the designated installation area involves diverse groups of stakeholders with diverging interests, make evaluating these socio-environmental impacts quite challenging. As an emerging technology with intrinsic uncertainties because of its newness, nearly no unclear policies exist related to its deployment. One of the rare examples of existing government policies is Vietnam’s regulations that offer more than 8% higher purchase price for electricity generated from FPV (USD 0.0769/kWh) than ground-mounted PV (Publicover, 2020). Even the government-supported policy in this case is limited to financial incentives only and does not reflect on socio-environmental or technical aspects of FPVs like water surface rent and if it could be used simultaneously for other actives, e.g., aquaculture, when possible. The global sustainable growth of FPVs market needs more than competitive power purchase prices and requires the development of extensive new regulations and policies. Developing such frameworks needs government-supported investments in research to address uncertainties related to the environmental impacts of FPVs, understanding the public perception of this technology, and addressing their concerns. Clarifying water rights for the owner and operator as well as accessibility to the FPVs if they are installed in restricted areas, including hydropower integrated systems, need to be addressed by clear regulatory processes.

Water bodies, regardless of their nature, e.g., a rainwater retention reservoir or lake behind a hydroelectric dam, intrinsically have dynamic nature because of the constant interaction with their surrounding environments. In the same way FPVs deployed on these water bodies are affected by external forces. Events like Typhoon Faxai that hit Japan in September 2019, although so rare that it can be considered as an exception and extreme scenario, still reveal a possible risk to FPVs. The 120-mph winds tore the modules off and stacked them on top of each other, which caused overheating and fire to break out, and Kyocera’s 13.7-MW floating solar power plant at the Yamakura Dam was damaged (Bellini, 2019). In early 2022, the 17-MW FPVs in Southern France experienced a fire accident, attributed to the FPVs exposure to several days of strong winds, causing repeated frictions of cables. Although this has been a minor incident, it shows the effects of wear and weather risks even in the short term (Beyer, 2022). If the fire spreads on FPVs, the combination of electricity, fire, and water makes tackling the blaze challenging. The other concern is the vulnerability of the floats to damage, which could cause such floating structures to sink. Such accidents and potential risks highlight the importance of investment and new supporting policies both in terms of financial compensations if such accidents happen and more importantly in research and development of FPVs. This is particularly crucial for offshore FPVs as they are exposed to harsh marine environment, while they could play a significant role in energy transition of the countries with land scarcity like the Netherlands.

### Conclusions

Floating solar farms are an emerging concept in clean energy, and a growing number of companies are working on different aspects of this technology, including float designs and materials. As an analogy, FPVs compared with ground-mounted solar are like offshore wind farms to onshore yet more cost-competitive. The recent crisis in Ukraine could also affect the development trajectory of both onshore and offshore FPVs. Issuing new drilling permits, softening restrictions on coal use, and subsidizing fossil fuels could affect the energy supply priorities in the short term. However, diversifying the energy supplies in the long run and developing roadmaps and frameworks for deploying emerging clean technologies could facilitate new investments in FPVs. FPVs could support countries to meet their energy trilemma and play a crucial role in the decarbonization of the energy sector and meeting the electricity demand growth. We could conclude that

• Reuse of degraded areas, mitigating land-use conflicts, more efficiency than the ground-mounted PV solar, saving water, improving resilience, and diversifying electricity-producing infrastructures are of FPVs key benefits.
• The existing technologies serve floating solar farms well, and no breakthrough is required to create exponential global growth in this market for inland waters.
• Financial concerns about the long-term performance and reliability of these systems, as well as the lack of supportive incentives provided by the governments, seem to be the main reason behind the slow development of large-scale FPVs.
• Creating new policies and frameworks for FPVs, which address social, environmental, and technical concerns of these systems, is essential for sustained growth of this technology.
• Deployment of offshore FPVs is far behind the FPVs that are placed on inland waterbodies. This is attributed to the stability and functioning risks that harsh marine environment poses on these systems, suggesting this area needs extensive further research.
• Despite the existing uncertainties and potential risks, the socio-environmental and technical opportunities that FPVs create make them an increasingly important part of the electricity supply.
