# Supplementary material for: Environmental and technical impacts of floating photovoltaic plants as an emerging clean energy technology
Source: iScience. 2022 Oct 4;25(11):105253. doi: 10.1016/j.isci.2022.105253 (PMC9587316; doi:10.1016/j.isci.2022.105253)
Supplement: Document S1. Tables S1–S3 and Figure S1 [file mmc1.pdf]

iScience, Volume *25*

Supplemental information

Environmental and technical impacts of floating photovoltaic plants as an emerging clean energy technology

Hamid M. Pouran, Mariana Padilha Campos Lopes, Tainan Nogueira, David Alves Castelo Branco, and Yong Sheng

Supporting Information

FPV system and Balbina Hydropower were compared to show some of the important technical factors involved, such as average annual energy generation, energy generated by occupied area, and a capacity factor of the two systems of 1 MW capacity.

The software System Advisor Model (SAM) was used to simulate the performance of the proposed FPV system installed in the reservoir of Balbina. The main technical characteristics of the proposed system are shown in Table S.1.

Table S.1: Main Technical Characteristics of Floating PV system

**Location and Resource**	
Latitude; Longitude	01°55′02″S 59°28′25″W
Global horizontal irradiation	5.13 kWh/m²/day
Average Temperature	25.4 °C
Average wind speed	0.1 m/s
**Module**	
Type	Globo Brasil - GRB Mono PERC
Module capacity	380 Wp
Efficiency	19.2%
Nominal operating temperature	45 °C
Temperature coefficient of max power point	-0.43%/°C
Module area	1.98 m²
**Inverter**	
Type	SMA America: SC750CP-US (with ABB EcoDry Ultra transformer)
Capacity	770,000 W
Efficiency	97.6%
**System Design of 1 MW**	
Number of inverters	1
DC to AC ratio	1.3
Modules per string in subarray	16
Strings in parallel in subarray	165
Orientation	Fixed, tilt 10°, Azimuth 0°
Ground coverage ratio (GCR)	0.49
Module area	5,227.2 m²
Total land area (1 MW)	10,512 m²
Module aspect ratio	1.98
**Losses**	
Soiling	0,01
Module mismatch	0,02
Diodes and Conections	0.5%
DC and AC wiring	2% and 1%
Transformer no load loss	1.1%

From the technical data of the proposed FPV system, System Advisor Model (SAM) software has calculated the following outputs: the annual energy generated (1,413 MWh for

the first year), the Capacity factor (16,1%), the Energy yield (1.408 kWh/kW) and the Performance ratio (0.75).

The Capacity factor of Brazilian Hydropower plants (HPP) can be calculated by dividing the average energy generation (based on data from Brazilian Nacional Operator of System (ONS, 2022)) by the installed capacity of all the Hydropower plants (CCEE, 2022). The result of average capacity factor of Brazilian hydropower plants in the period of 2013 to 2021 is shown in Table S.2. Figure S.1, also provides a graph to demonstrate the capacity factor of Balbina HPP in comparison with a hybrid Capacity factor of the integration of FPV system of 86 MWp and Balbina HPP.

Table S.2: Installed Capacity, Average MW generated and Capacity factor of Hydropower plants in Brazil

**Year**	**Installed Capacity of Brazilian HPP (MW) (CCEE, 2022)**	**Average MW generated in Brazilian HPP (ONS, 2022)**	**Capacity Factor**
2013	97,960	47,376	48.4%
2014	98,376	44,552	45.3%
2015	100,685	44,552	44.2%
2016	112,857	47,446	42.0%
2017	113,669	45,793	40.3%
2018	114,443	47,709	41.7%
2019	115,512	47,738	41.3%
2020	115,721	47,302	40.9%
2021	115,967	43,131	37.2%
	Average	46,178	42.4%

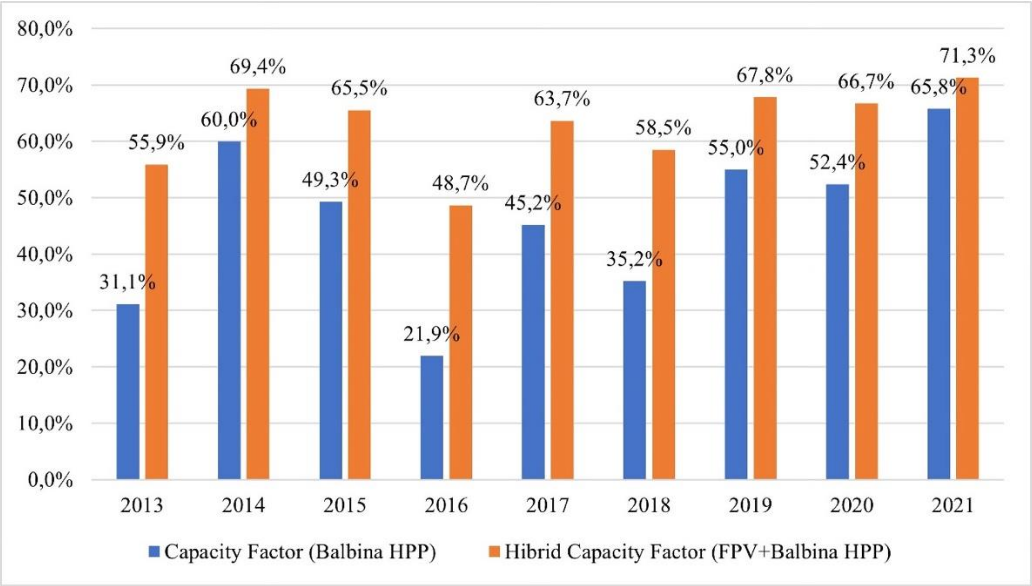

Figure S.1: Capacity factor of Balbina HPP in comparison with a hybrid Capacity factor of the integration of FPV system of 86 MWp and Balbina HPP.

The same data and methodology were used to calculate the Balbina's Capacity Factor. The result shows a value of 46.2% of Balbina Hydropower plant capacity factor, i.e., the substation and transmission line of Balbina have, in average, 53,8% of capacity idleness.

Another indicative compared was the annual energy generated divided by the occupied area for 1 MW of FPV and 1 MW of the Balbina hydropower. For this purpose, we considered that 1 MWp of FPV occupies 10,512 m² of the reservoir (Lopes et al., 2020) to produce the estimated energy of 1,408 MWh per year. For Balbina hydropower calculation it was considered that the energy production of 1,012,000 MWh (ONS, 2022) requires a reservoir area of 2,730 km² (ANA, 2019). The result shows that the annual energy generated by the occupied area is 133,9 kWh/m² for FPV plant in comparison of 0.37 kWh/m² for Balbina Hydropower. Efficiency in using space for power generation is almost 362 times higher in FPV systems compared to the Balbina hydropower plant.

Furthermore, the combined use of the FPV system in the Balbina reservoir could increase the capacity factor of the generation system. To calculate the impact of hybridization on the capacity factor, we can use the following equation:

$$CF_H = \frac{E_{FPV} + E_{HPP}}{Pot_{FPV} + APot_{HPP} \times 8760} \qquad \text{Equation 1}$$

Where, *CF* is the capacity factor for the hybrid generation (floating PV plus hydropower plant) in percentage; E_{FPV} is the annual energy generated by FPV system, in MWh; E_{HPP} is the annual energy generated by hydropower plant, in MWh; Pot_{FPV} is the installed power by floating PV system, in MW; $APot_{HPP}$ is the average power generated annually by hydropower plant in the MW, and 8760 is the number of hours in a year.

The calculation of $APot_{HPP}$ was based on the average annual power generated between 2013 to 2021. During these eight years, 2021 has provided the highest electricity output by hydropower plant, approximately 164 MW. A complementary FPV system was then proposed considering the average power delivered by the hydroelectric plant, in this case, 86 MW (Pot_{FPV}), so that two systems could reach the 250 MW predicted by the substation and transmission line.

The Capacity factors of Balbina HPP alone and after the proposed installation of FPV system of 86 MWp are shown in Table S.3.

Table S.3: Balbina's Capacity factor and the estimative of hybrid capacity factor with 85 MW FPV

Year	Balbina's power (average anual MW) (ONS, 2022)	Capacity Factor (Balbina HPP)	Balbina's Energy (MWh) (ONS, 2022)	Hibrid Capacity Factor (FPV+Balbina HPP)

2013	78	31.1%	682.000	55.9%
2014	150	60.0%	1313.000	69.4%
2015	123	49.3%	1079.000	65.5%
2016	55	21.9%	480.000	48.7%
2017	113	45.2%	989.000	63.7%
2018	88	35.2%	771.000	58.5%
2019	137	55.0%	1204.000	67.8%
2020	131	52.4%	1147.000	66.7%
2021	164	65.8%	1440.000	71.3%
**Average**	**115**	**46.2%**	**1011.667**	64.2%

References

{Bibliography}
